# Comparison of postoperative visual quality after SMILE and LASEK for high myopia: A 1-year outcome

**DOI:** 10.1371/journal.pone.0182251

**Published:** 2017-08-03

**Authors:** Xiaoyu Zhu, Leilei Zou, Manrong Yu, Chen Qiu, Minjie Chen, Jinhui Dai

**Affiliations:** 1 Department of Ophthalmology, Eye and ENT Hospital, Fudan University, Shanghai, People’s Republic of China; 2 Key Laboratory of Myopia, Ministry of Health, Shanghai, People’s Republic of China; University of Missouri-Columbia, UNITED STATES

## Abstract

**Purpose:**

To compare the 1-year outcome of visual quality after laser-assisted subepithelial keratomileusis (LASEK) and femtosecond laser-assisted small incision lenticule extraction (SMILE) for high myopia correction.

**Materials and methods:**

This prospective, comparative study included 24 eyes of 24 patients in the LASEK group, with a mean spherical equivalent (SE) of -7.59 ± 1.32 diopters, and 26 eyes of 26 patients in the SMILE group, with a mean SE of -7.91 ± 1.08 diopters. Visual acuity, corneal topography, contrast sensitivity (CS), and wavefront aberrations were recorded preoperatively and compared with postoperative measurements. Objective scatter index (OSI) and modulation transfer function (MTF) cut-off frequency were measured 1 year postoperatively.

**Results:**

One year postoperatively, the two groups demonstrated no significant difference in the CS at all spatial frequencies. The increments of higher-order aberrations (HOAs) (HOA = 0.583 ± 0.210 μm), including spherical aberration (SA) (SA = 0.546 ± 0.249 μm), were higher (P < 0.05) in the LASEK group than those in the SMILE group (HOA = 0.451 ± 0.143 μm; SA = 0.450 ± 0.340 μm) after surgery. There were no significant differences in the increments of coma and trefoil aberrations between the two groups. The OSI and MTF cut-off frequency exhibited no significant differences between the two groups postoperatively. No vision-threatening complications were noted at any stage in either group.

**Conclusions:**

Both LASEK and SMILE are safe and effective surgical options for the correction of high myopia. SMILE has a lower HOAs and SA induction rate 1 year postoperatively.

## Introduction

Myopia is the most common ocular disorder that causes visual dysfunction. Myopia was reported to affect approximately 1406 million people worldwide in 2000, among whom163 million exhibited high myopia (2.7% of the world population)[1]. As the prevalence of myopia is still increasing yearly, some scholars predict that, 49.8% and 9.8% of the world’s population will have myopia and high myopia, respectively, by the year 2050[1]. Uncorrected refractive error has brought and will bring an increasing financial and social burden per annum[2].

Therefore, additional techniques have been introduced to correct refractive errors. In 1998, laser-assisted subepithelial keratomileusis (LASEK) became a priority surgical choice to correct refractive errors. With rapid development, small incision lenticule extraction (SMILE) emerged as the latest generation technology in 2008. Several studies have proved that LASEK and SMILE are both safe, effective and predictable[3,4].

With the improvement in refractive surgery, more concern has transferred from safety and efficacy to postoperative visual quality. However, all corneal refractive surgeries will inevitably elevate the risk of postoperative glare, haloes and night vision defects because they change the natural path of light to the retina. These changes are even more pronounced in patients with high myopia due to the thicker ablation depth/lenticule thickness.

The postoperative higher-order aberrations (HOAs) of LASEK are lower than those of laser in situ keratomileusis (LASIK), which requires a flap, whereas LASEK does not[5]. Also, thanks to the flapless and other minimally invasive features, SMILE, an all-femtosecond laser refractive procedure, had been proven to yield better visual quality than LASIK[6]. However, to our knowledge, studies comparing the long-term visual quality between LASEK and SMILE with respect to correcting high myopia are still needed. Therefore, the aim of the present investigation was to compare the 1-year outcome of visual quality in patients undergoing refractive surgery using SMILE or LASEK to correct high myopia, including wavefront aberrations, contrast sensitivity (CS), objective scatter index (OSI) and modulation transfer function (MTF) cut-off frequency.

## Materials and methods

### Participants

This was a prospective, non-randomized, comparative study, involved 46 eyes of 24 patients (9 males and 15 females) who underwent LASEK and 50 eyes of 26 patients (7 males and 19 females) who underwent SMILE from January 2013 to January 2015 at the Eye and ENT Hospital of Fudan University, Shanghai, People’s Republic of China. Only the data from the left eye were used for the statistical analysis. The main inclusion criteria were as follows: age between 18 and 45 years, spherical equivalent (SE) from -6.00 to -10.00 D, a stable refractive error for at least 2 years (≤-0.25 diopters [D] change each year), minimum corneal thickness above 480 μm, best corrected distant visual acuity (CDVA) of 20/25 or better, and no systemic or localized ocular disease.

This study was approved by the institutional review board of The Ethics Committee of the Eye and ENT Hospital of Fudan University. All patients provided written informed consent before the surgery, and they were treated in accordance with the tenets of the Declaration of Helsinki.

### Preoperative examinations

All patients underwent the routine preoperative examinations for refractive surgery, including measurements of CDVA, slit-lamp examination, intraocular pressure (IOP) with non-contact tonometry, refraction (manifest and cycloplegic), fundus examination with a three-mirror contact lens, corneal topography (Pentacam; Oculus Optikgeräte, Wetzlar, Germany), wavefront aberrations (WASCA wavefront analyzer; Carl Zeiss Meditec AG, Jena, Germany) and CS (Takagi Contrast Glare Tester CGT-1000; Takagi Seiko Co. Ltd., NaganoKen, Japan).

### Surgical techniques

All the surgeries were performed by the same surgeon. The target of all postoperative refraction was emmetropia.

The LASEK surgical procedure began with a 14-second 20% ethanol-assisted epithelial removal, followed by a standard excimer laser ablation using the Mel-80 excimer laser (Carl Zeiss Meditec AG) with a repetition rate of 250 kHz and a pulse energy of 150 nJ. The optical zone diameter ranged from 6.00 to 6.50 mm, and the transition zone diameter ranged from 7.5 to 8.0 mm. The LASEK was performed under the tissue-saving ablation profile and with a 30-second 0.02% mitomycin. Then, the epithelium was repositioned after laser ablation, and a bandage contact lens (ACUVE OASYS; Johnson & Johnson, New Brunswick, NJ) was applied for 7 days.

SMILE was performed using the Visumax femtosecond laser system (Carl Zeiss Meditec AG) with a repetition rate of 500 kHz and a pulse energy of 130 nJ. The cap thickness was targeted to range from 110 to 120 μm, with an intended diameter of 7.6 mm. The lenticule diameter was set from 6.0 to 6.6 mm. The refractive lenticule of the intrastromal corneal tissue was dissected and then removed through a superior incision opening, 2 mm in length, using surgical forceps[7].

Postoperatively, levofloxacin 0.5% eye drops (Santen Pharmaceutical Co., Ltd.) were used four times daily for 7 days. Artificial tears (Hypromellose 2910, dextran 70, glycerol eye drops; Alcon Laboratories, Inc., Fort Worth, TX) were used four times daily for 90 days. Fluorometholone 0.1% eye drops (Santen Pharmaceutical Co., Ltd.) were initially used six times daily and then were tapered for a period of 60 days for LASEK and 30 days for SMILE.

### Postoperative ophthalmologic examinations

All postoperative patients were followed up regularly. Examinations, including measurements of uncorrected visual acuity (UCVA), CDVA, slit-lamp examination, IOP with non-contact tonometry, refraction with an auto-refractometer, corneal topography, wavefront aberrations, CS, OSI and MTF cut-off frequency (OQASII, Visiomereics SL, Spain), were scheduled for 1 year after the surgery.

Ocular wavefront aberrations, including the Zernike coefficients of vertical trefoil, horizontal trefoil, vertical coma, horizontal coma, spherical aberration (SA) and the root mean square (RMS) of coma, trefoil and HOAs were analyzed with a standardized pupil diameter of 6 mm.

CS was measured with and without glare at six target sizes: 6.3°, 4.0°, 2.5°, 1.6°, 1.0°, and 0.7° which have 13 contrast levels(2.00 to 0.34) with an average step size of 0.15 log_10_CS.

Based on a double-pass technique, the OSI and MTF cut-off frequency were measured to display the objective image quality on the retina. All measurements were conducted in mesopic conditions with a 4.0-mm artificial pupil and the results were calculated using the average values of three measured parameters.

### Statistical analysis

The data analysis was performed using SPSS19.0 software (SPSS, Inc., Chicago, IL). All data were reported as the mean ± standard deviation. We used a t-test to compare the normally distributed data between two groups and a paired t-test to compare the data before and after each surgery. These non-normally distributed data were analyzed by the Mann-Whitney rank-sum test and Wilcoxon signed-rank test. A P value < 0.05 was considered statistically significant.

## Results

Forty-six eyes of 24 patients underwent LASEK, 50 eyes of 26 patients underwent SMILE, and these patients completed all of the pre- and postoperative examinations. There was no significant difference in the average age between the two groups (t = 1.284, P = 0.205), and the results of the preoperative examinations between the two groups were comparable (Table 1).

**Table 1:** Preoperative demographic data^a^.

Parameter	LASEK (n = 24)	SMILE (n = 26)	t	P
Age (y)	30.38 ± 7.13	27.77 ± 7.21	1.284	0.205
CDVA (logMAR)	0.010 ± 0.049	0.033 ± 0.045	-1.735	0.089
Spherical equivalent (D)	-7.59 ± 1.32	-7.91 ± 1.08	0.946	0.349
Sphere (D)	-7.17 ± 1.17	-7.51 ± 1.02	1.106	0.274
Cylinder (D)	-0.85 ± 0.69	-0.81 ± 0.54	-0.267	0.791
CCT (μm)	544.13 ± 31.55	547.81 ± 26.49	-0.448	0.656
Ablation depth/Lenticule thickness (μm)^b^	131.83 ± 12.81	144.31 ± 11.08	-3.69	0.001
IOP (mmHg)	15.31 ± 2.69	15.51 ± 2.30	-0.288	0.775
Scotopic pupil diameter (mm)	7.23 ± 0.57	7.00 ± 0.65	1.27	0.210
Vertical coma (μm)	0.026 ± 0.401	-0.017 ± 0.348	0.404	0.688
Horizontal coma (μm)	-0.048 ± 0.368	-0.074 ± 0.426	0.227	0.822
Coma (μm)	0.489 ± 0.222	0.502 ± 0.214	0.212	0.833
Vertical trefoil (μm)	0.019 ± 0.248	-0.027 ± 0.320	0.570	0.571
Horizontal trefoil (μm)	-0.126 ± 0.353	-0.121 ± 0.312	-0.049	0.961
Trefoil (μm)	0.388 ± 0.214	0.376 ± 0.263	0.182	0.856
Spherical aberration (μm)	0.256 ± 0.170	0.274 ± 0.227	-0.313	0.756
HOA (μm)	0.301 ± 0.181	0.309 ± 0.208	-0.151	0.88

LASEK = laser-assisted subepithelial keratomileusis; SMILE = small incision lenticule extraction; CDVA = corrected distance visual acuity; D = diopters; CCT = central corneal thickness; IOP = intraocular pressure; HOA = higher-order aberrations.

^a^Mean values were expressed as mean ± standard deviation.

^b^P values < 0.05.

### Efficacy and safety

The UCVAs of LASEK and SMILE were both improved 1 year after surgery, but there was no significant difference between the groups. In total, 79.2% of the treated eyes in the LASEK group and 76.9% in the SMILE group attained a UCVA of 1.0 (20/20 Snellen) or better. The efficacy indices did not significantly differ between the two groups. In the LASEK group, 37.5% of the treated eyes had an unchanged CDVA, 45.8% gained one line, 12.5% gained two lines, and 4.2% lost one line. In the SMILE group, 30.8% of the treated eyes had an unchanged CDVA, 61.5% gained one line, and 7.7% gained two lines. The safety indices did not significantly differ between the groups. All surgical procedures were uneventful. There were no serious intraoperative or postoperative complications, and no haze occurred in the LASEK group 1 year postoperatively (Table 2).

**Table 2:** Demographic data one year postoperatively^a^.

Parameter	LASEK (n = 24)	SMILE (n = 26)	t	P
UCVA (logMAR)	0.009 ± 0.139	0.020 ± 0.093	-0.321	0.750
CDVA(logMAR)	0.075 ± 0.064	0.104 ± 0.064	-1.630	0.110
Efficacy Indices	1.04 ± 0.28	1.00 ± 0.22	0.603	0.550
Safety Indices	1.17 ± 0.17	1.19 ± 0.14	-0.313	0.756
Spherical equivalent (D)	-0.516 ± 0.418	-0.327 ± 0.314	-1.814	0.076
CCT (μm)^b^	447.17 ± 30.63	426.69 ± 27.02	2.511	0.015
IOP (mmHg)	9.48 ± 2.13	10.07 ± 2.75	-0.849	0.400

LASEK = laser-assisted subepithelial keratomileusis; SMILE = small incision lenticule extraction; UCVA = uncorrected visual acuity; CDVA = corrected distance visual acuity; D = diopters; CCT = central corneal thickness; IOP = intraocular pressure.

^a^Mean values were expressed as mean ± standard deviation.

^b^P values < 0.05.

### Refraction and central corneal thickness

The postoperative SE did not significantly differ between the groups. The postoperative central corneal thickness (CCT) was greater in the LASEK group than in the SMILE group. The postoperative IOP showed no significant differences between the two groups (Table 2).

### Wavefront aberrations

**Fig 1:**
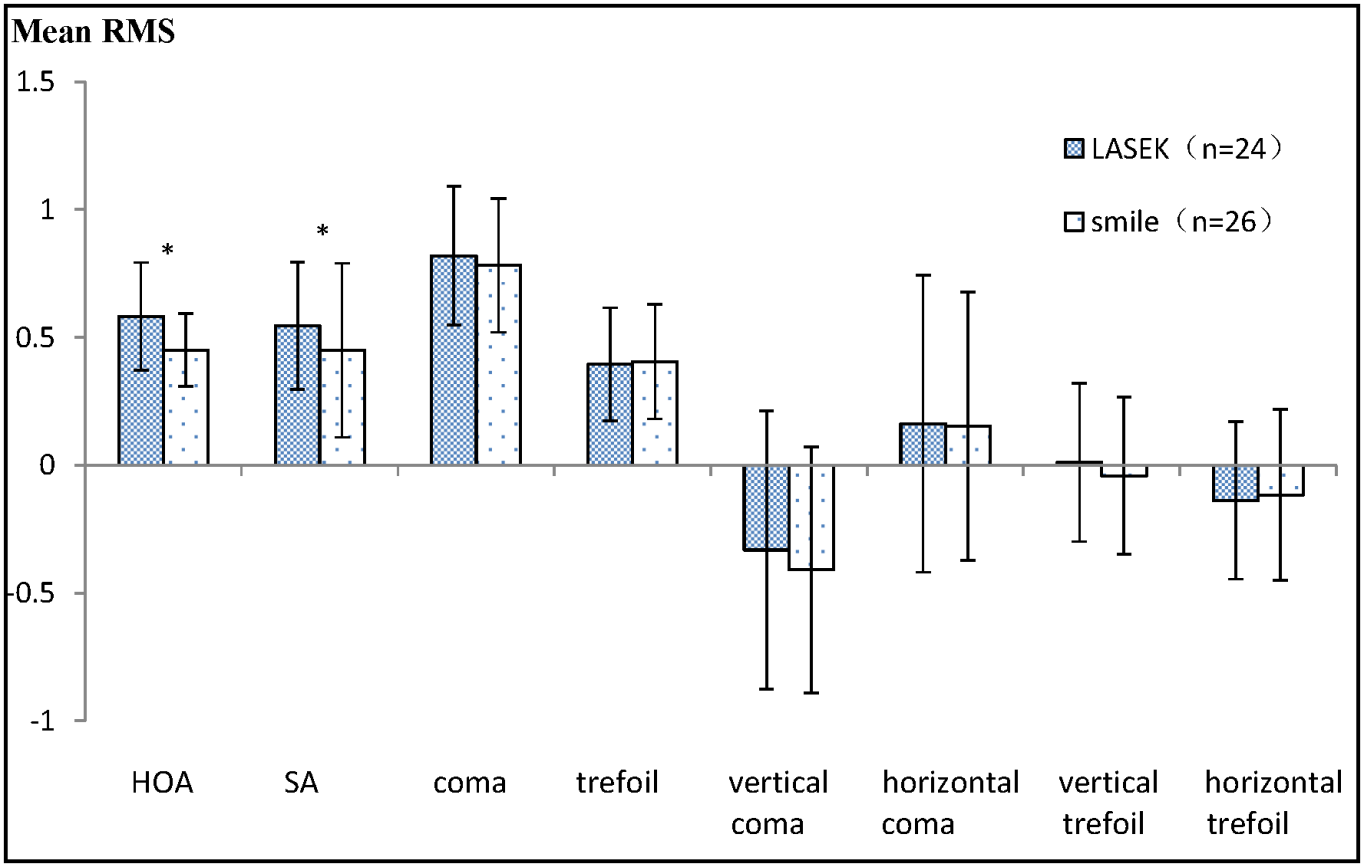
Comparison of the wavefront aberrations between laser-assisted subepithelial keratomileusis (LASEK) and the small incision lenticule extraction (SMILE) groups 1 year after surgery. *P < 0.05 = statistically significant. HOA = higher-order aberrations; SA = spherical aberration; RMS = root mean square.

There were no significant differences between the LASEK and SMILE groups with respect to the preoperative RMS of aberrations, including HOA, SA and coma (Table 1). The comparison of the pre- and post-operative parameters demonstrated that HOA, SA and coma increased significantly in both the LASEK and SMILE groups (paired t-test; LASEK group: HOA, t = -6.084, P < 0.001; SA, t = -7.902, P < 0.001; coma, t = -5.893, P < 0.001; SMILE group: HOA, t = -3.104, P = 0.005; SA, t = -4.908, P < 0.001; coma, t = -6.411, P < 0.001). There were no significant differences before and 1 year after surgery in trefoil aberrations (LASEK: t = -0.339, P = 0.737; SMILE: t = -1.056, P = 0.301). One year postoperatively, the increments of HOAs and SA were higher in the LASEK group than in the SMILE group (ΔHOA: t = 2.155, P = 0.036; ΔSA: t = 2.214, P = 0.032). The increment of coma aberrations was higher in the LASEK group than in the SMILE group, but there was no significant difference between these two groups (Δcoma: t = 0.722, P = 0.473). No significant difference in the increment of trefoil aberrations (t = -0.698, P = 0.489) was found between the groups (Fig 1).

### Contrast sensitivity

**Fig 2:**
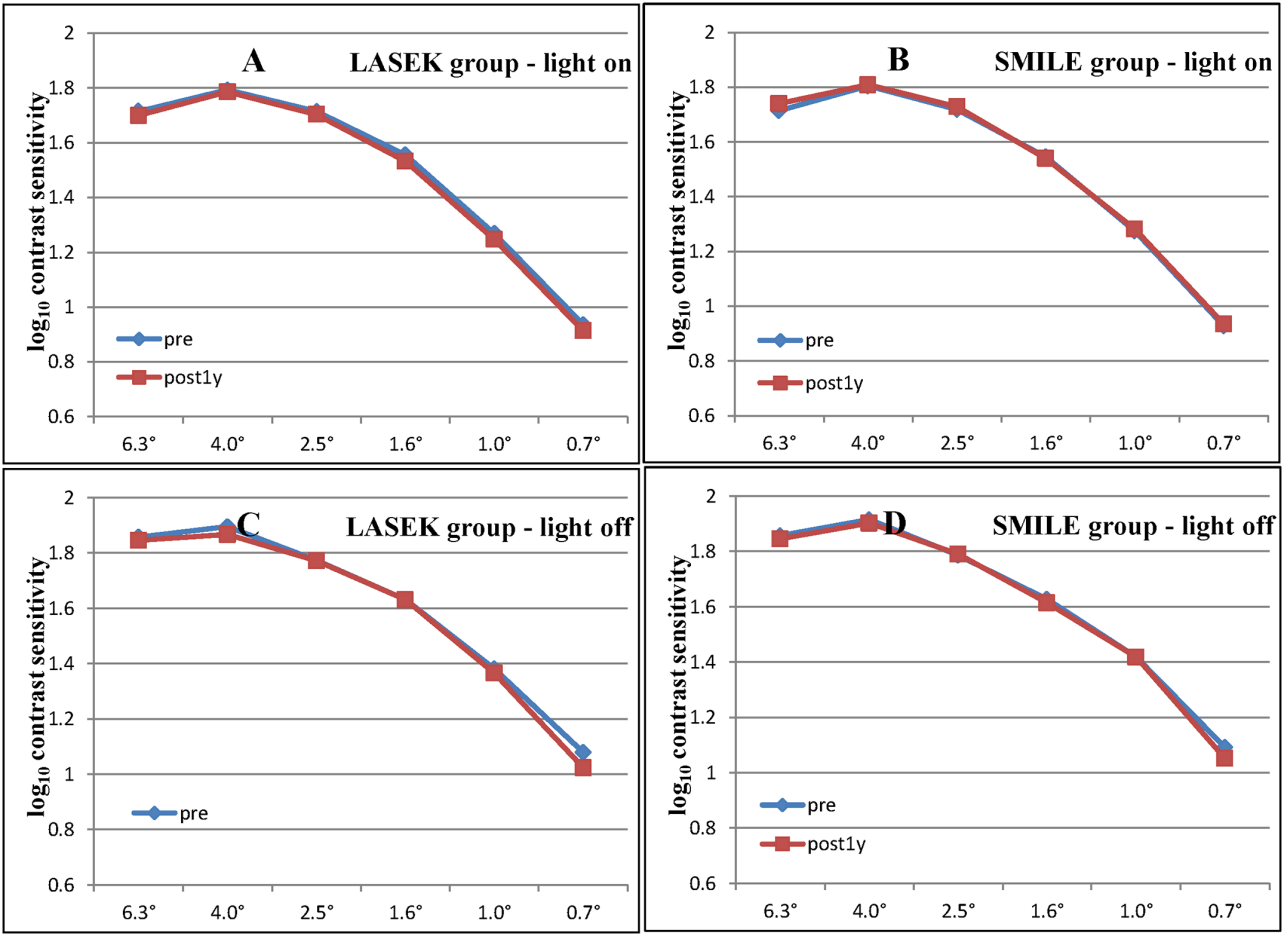
Comparison of (A, B) photopic and (C, D) scotopic contrast sensitivity between the (A, C) laser-assisted subepithelial keratomileusis (LASEK) and the (B, D) small incision lenticule extraction (SMILE) groups at all spatial frequencies (6.3° to 0.7°), preoperatively and postoperatively. Horizontal axis corresponds to visual angle of target size (degree). Vertical axis corresponds to log_10_ contrast sensitivity.

Preoperatively, there were no significant differences between the LASEK group and the SMILE group regarding the photopic and scotopic CS at six spatial frequencies (Table 3). No significant difference in CS was detected before and 1 year after surgery in both groups at all spatial frequencies. One year postoperatively, the decrements in CS between the two groups across all spatial frequencies were not significantly different (Fig 2).

**Table 3:** Preoperative contrast sensitivity (log_10_)^a^.

Contrast sensitivity	LASEK (n = 24)	SMILE (n = 26)	t	P
Light on 6.3°	1.715 ± 0.117	1.714 ± 0.130	0.029	0.977
4.0°	1.793 ± 0.122	1.804 ± 0.114	-0.332	0.741
2.5°	1.714 ± 0.122	1.718 ± 0.147	-0.125	0.901
1.6°	1.556 ± 0.102	1.545 ± 0.132	0.303	0.763
1.0°	1.269 ± 0.247	1.275 ± 0.162	-0.103	0.919
0.7°	0.935 ± 0.199	0.927 ± 0.157	0.147	0.884
Light off 6.3°	1.858 ± 0.113	1.857 ± 0.119	0.034	0.973
4.0°	1.895 ± 0.103	1.915 ± 0.096	-0.699	0.488
2.5°	1.773 ± 0.154	1.786 ± 0.127	-0.309	0.759
1.6°	1.631 ± 0.141	1.627 ± 0.167	0.092	0.927
1.0°	1.381 ± 0.142	1.412 ± 0.158	-0.862	0.393
0.7°	1.079 ± 0.165	1.092 ± 0.172	-0.269	0.789

LASEK = laser-assisted subepithelial keratomileusis; SMILE = small incision lenticule extraction.

^a^Mean values were expressed as mean ± standard deviation.

### OSI and MTF cut-off frequency

One year postoperatively, although the results of the OSI and the MTF cut-off frequency were better for SMILE than for LASEK, there were no significant differences between LASEK and SMILE regarding both OSI (LASEK = 1.275 ± 1.080, SMILE = 1.065 ± 0.524, t = 0.884, P = 0.381) and MTF cut-off frequency (LASEK = 29.846 ± 9.023cpd, SMILE = 32.697 ± 9.072cpd, t = -1.113, P = 0.271).

## Discussion

Kulkarni et al. reported 1-year visual and refractive outcomes of LASEK[8]. It was found that 84% of the treated eyes in the high myopia group attained a UCVA of 20/20 or better and that 60% achieved a CDVA of 20/15. The mean postoperative SE was below -0.5 D postoperatively, and no serious complications occurred. Kim et al. reported that[9], one year after SMILE, 78.4% of the treated eyes in the high myopia group attained a UCVA of 20/20 or better, 43.2% had an unchanged CDVA, 47.2% gained one line, and 6.4% gained two lines. The mean postoperative SE was -0.25 ± 0.35D postoperatively and no visually threatening complications occurred.

In our study, both LASEK and SMILE were proved effective, with no significant difference observed between the CDVA before the operations and the UCVA achieved after. In addition, all of the patients attained a UCVA of 10/20 or better postoperatively, with 79.2% and 76.9% of the treated eyes in the LASEK and SMILE groups, achieving a UCVA of 20/20 or better, respectively. Furthermore, both the LASEK and the SMILE groups gained a better postoperative CDVA compared with the preoperative CDVA, with safety indices of 1.17 ± 0.17 and 1.19 ± 0.14 in the LASEK and SMILE groups, respectively. Consistent with previous studies[4,8,9,10], our findings showed that both LASEK and SMILE are safe and effective.

Yu et al. reported that there are no significant differences in UCVA between SMILE and LASEK in patients with mild to moderate myopia 3 months postoperatively[11]. The current study, which showed that there were no significant differences in the postoperative UCVA, efficacy index, safety index and SE between LASEK and SMILE 1 year postoperatively, suggests that LASEK is equally effective and safe as SMILE for correcting high myopia.

The differences in the ablation depth/lenticule thickness and postoperative CCT of the present study between the two groups showed that the lenticule thickness of SMILE was thicker than the ablation depth of LASEK. This phenomenon may due to the distinction between the laser types. The VisuMax femtosecond laser needs to remove a greater amount of corneal tissue than does the MEL-80 excimer laser for correction per 1.0D of myopia[11,12,13]. Nevertheless, there was no significant difference in the SE between the two groups.

With the development of refractive surgery, researchers have shifted more attention from efficacy and safety to visual quality postoperatively. It is well known that visual quality of human eye can be affected by several factors, such as tear film stability, corneal shape, wavefront aberration, pupil size, lens density, etc. Until now, no matter what kind of refractive surgery, have the potential to change the shape of cornea, raise the wavefront aberration and damage the tear film stability, Thereby, causing a potentially deleterious effect on the postoperative visual quality[14,15], and resulting in symptoms such as glare and haloes. However, there was still no reported clinical study comparing the visual quality after SMILE and LASEK for correcting high myopia, let alone the long-term difference. Therefore, we measured visual quality parameters including wavefront aberration, CS, OSI and MTF cut-off frequency 1 year after SMILE and LASEK to determine which surgery is the better choice for the long-term visual quality when correcting high myopia.

In a comparative study, Ganesh et al. found that postoperative HOAs were significantly fewer in the SMILE group than in the FS-LASIK group, whereas both operations caused an increase in HOAs[16]. McAlinden et al. attributed postoperative aberrations induced by LASIK rather than LASEK to the flap creation[17]. Benefiting from the flapless characteristic, both LASEK and SMILE induced fewer HOAs than LASIK[5,17].

Our results of wavefront aberration showed that HOAs, especially SA, increased more significantly 1 year after LASEK than after SMILE, while HOAs, including SA and coma aberrations, increased significantly in both the LASEK and the SMILE groups postoperatively, which was also observed by Yu et al. in their 3-month study[11].

HOAs are the crucial factors in determining the visual quality after refractive surgery. Among them, SA plays a more important role in visual quality than do coma-like aberrations, particularly under a large pupil diameter[18]. Therefore, increased HOAs, especially SA after surgery, may elevate the potential risk of glare and haloes in dark environments. Regardless, LASEK or SMILE both flatten the cornea, thus raising aberrations. Furthermore, postoperative refractive reduction and corneal remodeling associated with the incision healing process, may increase HOAs[19]. As it induces a lower incision-healing response and is an all-in-one femtosecond laser procedure, SMILE can minimize changes in the corneal shape[20], thus resulting in fewer HOAs. Our results demonstrated that SMILE, through a lower wavefront aberration induction, may achieve a better visual quality than LASEK for the correction of high myopia.

With regard to CS, our results revealed no significant difference before and after surgery in both LASEK and SMILE at all spatial frequencies. Moreover, there was no significant difference between the two groups postoperatively across all spatial frequencies.

Yu et al. reported that both SMILE and LASEK cause a slight reduction in high spatial frequency CS 1 month after surgery, which is recovered 3 months postoperatively[11]. Townley et al. found no significant CS changes occurred in the LASEK group under photopic or scotopic conditions 1 year postoperatively[21]. A study by Tan et al. looking at the outcomes following SMILE showed that both mesopic and photopic CS decreased significantly 3 months after surgery at higher frequencies but was recovered by 1 year[22].

Contrast sensitivity, defined as the ability to detect the minimal disparity in luminance between two objects, was considered a better index of visual quality than visual acuity. A few studies have shown that CS declines after corneal refractive surgery but recovers to preoperative levels within 3 months to 1 year after surgery[11,22]. LASEK and SMILE are both prone to raised wavefront aberrations and instability of the tear film during the early postoperative stage[11,23,24]. Over time, owing to restability of the tear film and a gradually restored elevation of wavefront aberration, long-term CS will rise again. Liou et al. found that, compared with contact lens correction, spectacle lens reduced the CS when correcting high or severe myopia, and they attributed this finding to the image shrink caused by the vertex distance effect[25]. The statistically unchanged CS of the present study occurred because of either the postoperative recovery effect within 1 year, or the lower preoperative CS caused by the examined spectacle lens correction, or even both. Based on this result, regardless of the reason, patients with high myopia were unaffected in CS after both LASEK and SMILE.

Intraocular scattering also plays an important role in visual quality and might reduce the contrast of the retinal image, particularly in patients with refractive medium opacity and after refractive surgeries[26,27]. Lee et al. reported that intraocular scattering is a reliable predictor for retinal image quality after LASEK and LASIK[28]. Based on a double-pass system technique, the OQASII system is the objective, quantitative and repeatable instrument for image quality measurements of the retina[29] and has been used in optical quality evaluation after several corneal refractive surgeries[28,30]. Therefore, it was applied to compare the visual quality postoperatively.

Our results showed that there were no significant differences between the two groups regarding both OSI and MTF cut-off frequency, although these values were both better in SMILE than in LASEK.

The OSI is an index reflecting the intraocular scattered state of light[31]. The MTF cut-off frequency represents the highest spatial frequency at the lowest contrast (1% of contrast) while the MTF reaches a value of 0.01[31]. Lee et al. reported that the OSI and MTF cut-off frequency were not significant correlated with ocular aberrations[28]. Ondategui et al. compared photorefractive keratectomy (PRK) and LASIK and concluded that corneal refractive surgery may deteriorate these values postoperatively[15]. They also found a significant correlation between OSI and achieved refractive correction and thus attributed the increment of ocular scattering to the ablation procedure rather than to flap creation after refractive surgery. Miao et al. reported that SMILE caused an increment in the OSI 20 days after surgery, which was gradually recovered 3 months postoperatively[30]. The refractive corrections of the present study were comparable between the two groups. Therefore, after a 1-year postoperative recovery, the OSI and MTF cut-off frequency after LASEK were not significantly worse than those after SMILE when correcting high myopia.

One limitation of the present study is that we did not measure the preoperative baseline of the OSI and MTF cut-off frequency. However, there were no significant differences in the preoperative CDVA, achieved refractive correction and other important refractive values between the two groups. Furthermore, the surgical patients had no serious ocular disease, such as cataract, which could affect the refractive media. Considering that the OSI and MTF cut-off frequency were associated with these factors[15,29], these values were likely to be comparable between the two groups preoperatively.

In conclusion, the current 1-year study indicates that both LASEK and SMILE are excellent surgical options for correcting high myopia. When considering all the visual quality measurements, SMILE may be a superior option over LASEK in providing a painless, high-refractive-accuracy procedure that subsequently yields better visual quality. A larger sample size and a visual-quality questionnaire will be employed in further research.
